# A Retrospective Analysis of Clinical and Pathologic Characteristics of Recurrent Bladder Tumor in a Tertiary Hospital in Addis Ababa, Ethiopia

**DOI:** 10.4314/ejhs.v31i4.12

**Published:** 2021-07

**Authors:** Andualem Beyene

**Affiliations:** Dept. Surgery, School of Medicine, College of Health Sciences, Addis Ababa University

**Keywords:** Bladder Tumor, Recurrence, Clinical Character, Pathologic character

## Abstract

**Background:**

Previous recurrences, tumor category (Ta, T1), the presence of CIS with superficial bladder tumors, the number of tumors, tumor size and multiplicity are predictors of bladder tumor recurrence. Recurrence is better predicted by multiplicity, size and previous recurrence.

**Methods:**

This is retrospective descriptive study. All patients with urinary bladder tumor admitted to TASH from January 1st, 2018 to December 31st, 2019 is the study population. Information was retrieved using structured questionnaire.

**Results:**

Most of the recurrent tumors 55 (76.4%) had huge size and were multiple 62 (86.1%) in the primary presentation. Most recurrent tumors 47 (65.3%) are low grade bladder tumors. About 17 (23.6%) were high grade tumor in their primary presentation. European studies showed size and multiplicity increase risk of recurrence. However, our patients have late presentations which probably made the proportion of recurrence higher.

**Conclusion:**

Most of the recurrent bladder tumors have huge size and multiple in number at their initial presentation. All histological variants of Urothelial carcinomas recur.

## Introduction

Bladder tumor is the second commonest tumors of the urinary tract (1). World-wide it has different incidence in males and females (7^th^ in males and 14^th^ in females) and it ranks 5^th^ in cancer occurrence in the USA (2,3). The most common type of bladder tumor is histologically transitional cell carcinoma (4). Transitional carcinoma of the bladder is classified as non-muscle invasive tumor (NMIBT) and muscle invasive tumor (MIBT). The most common type of transitional cell carcinoma is NMIBT which is about 75% (5).

Painless hematuria is the hallmark of bladder tumor. The hematuria is usually terminal hematuria which could become throughout as the disease progresses. Bladder tumor can also present with lower urinary tract symptoms (LUTS) and suprapubic pain. At a later stage it can obstruct the ureters and may present with loin pain and signs of renal failure (6).

This classification determines the modality of treatment. Whereas NMIBTs are treated with transurethral resection of bladder tumors (TURBT) and intravesical therapies, muscle invasive tumor requires radical cystectomy, radiotherapy and systemic chemotherapy (7). TURBT is the gold standard treatment for NMIBT (8). TURBT is the Gold standard treatment for NMIBT and carcinoma in situ tumors. It is both diagnostic and therapeutic. (9).

Bladder tumor after TURBT can recur and/or progress. Recurrence is occurrence of the same grade and stage tumor after complete resection while progression is a change from a NMIBT to MIBT (8,10).

Bladder tumor is a recurrent tumor at a rate of 15% to 70% in the first year (11). Factors associated with bladder tumor recurrence are size, multiplicity, tumor category (Ta, T1) CIS and superficial bladder tumor, grade of tumor and previous recurrence. Bladder tumor size of greater than 3 centimeters, tumors more than one and high-grade histology predict the likelihood of recurrence. Recurrence of more than once in a year is also predictive of recurrence (11, 12,13).

Many studies describe several predictors of bladder tumor recurrence. These include patient demographics (Age, sex) and tumor characteristics. The different tumor characters include size, grade, multiplicity, previous recurrence and T category and CIS. These tumor characters are put together in a scoring system out of 17 and patients are sorted in different risk categories. The categories are low risk, intermediate risk and high risk (14).

Tumor size is segregated in to tumor less than 3cms and greater than or equal to 3cms. And it is scored as 0 or 3. Tumor grades are given grades 1, 2, or 3 based on the World Health Organization 2004 classification. Grade 1 is UNLMP while grade 2 and 3 are low grade and high-grade tumors, respectively (15, 16,17). The main objective of the study is to describe clinical and pathologic characteristics of recurrent bladder tumor. It is also to identify the demography of patients and to describe pathologic characters of recurrent bladder tumor.

Bladder tumor is one of the common tumors treated at Tikur Anbessa Specialized Hospital (TASH). Most of the patients undergo TURBT and in one survey it accounts for 14.1% of all urologic procedures in the hospital (18). Some of the patients come with recurrent bladder tumors.

Post-TURBT patients are kept on follow-up to detect recurrence early and treat accordingly. Protocols of follow-up vary from centers to centers. There is no follow up protocol in TASH. However, it is wise to follow patients with higher probability of recurrence frequently with short intervals. This helps to schedule patients at different time tables depending on their risk of recurrence. This reduces hospital burden and early detection of recurrence. Therefore, this study helps to identify patient characteristics and develop a follow up protocol for patients with recurrent bladder tumor at TASH.

## Methods

This is a retrospective study to assess clinical and tumor characteristics of recurrent bladder tumor in TASH from January 1^st^, 2018 to December 31^st^, 2019. The study site is Tikur Anbessa Specialized Hospital. Data was collected from patient records on a predesigned questionnaire prepared by modifying and incorporating the European Organization for Research and Treatment of Cancer (EORTC) risk tables. We tried to describe patient demography, clinical conditions, tumor characters and treatment protocols. The study population is all patients with recurrent bladder tumor. Sampling is done with 95% confidence interval and 0.05 margin of error. A finite source population the period is taken to be 316. We took a sample size of 177 patients randomly for the study.

Data was entered in SSPS version 21 statistical program. The data was analyzed for patient demography, clinical and diagnostic parameters. In addition, we tried to make a descriptive assessment of tumor characteristics in recurrent bladder tumor patients. Patients with NMIBT, recurrent bladder tumor and biopsy (histological) proven are included in the study. Patients with MIBT and for whom biopsy not done are excluded. The study obtained ethical approval by the surgical department research and publication committee and College of Health Sciences institutional review board.

**Operational definitions:**

Non-muscle invasive tumor: Bladder tumor that has not involved the muscularis propria of the bladder

Recurrent bladder tumor: non-muscle invasive tumor that has appeared again after complete removal by transurethral resection.

Patients: are persons admitted to TASH and who had undergone surgical treatment for bladder tumor

This is a single hospital based retrospective study. This is a limitation; however, this study is going to be done at the main referral hospital in the country and the information from this study contribute a lot to feel the existing information gap and it will be used as base line study to do further country wide study.

## Results

We retrieved the medical records of 177 patients. Seven of them have incomplete data record and we excluded them. Of the 170 patients 129 (75.9%) males and 41 (24.1%) females. When we stratify by age 19 (11.2%) are less 40 years, 99 (58.2%) between 40 and 60 years and 52 (30.6%) above 60 years. Most patients 58 (32.6%) are from Oromia region followed by Amhara region and Addis Ababa city accounting 42 (23.7%) each (Table 1).

**Table 1:** Demographic characters of patients admitted to TASH (n=170)

Variables and category		Frequency	Percent
Age	<40	19	11.2
	40–60	99	58.2
	>60	52	30.6
	Total	170	100
Sex	Male	129	75.9
	Female	41	24.1
	Total	170	100
Address	Oromia	58	34.1
	Addis	42	24.7
	Ababa		
	Amhara	42	24.7
	*SNNPR	17	10.0
	Harari	4	2.4
	Dire Dawa	3	1.7
	Not	4	2.4
	recorded		
	Total	170	100

*SNNPR: Southern Nations, Nationalities and Peoples Region

**Figure 1:**
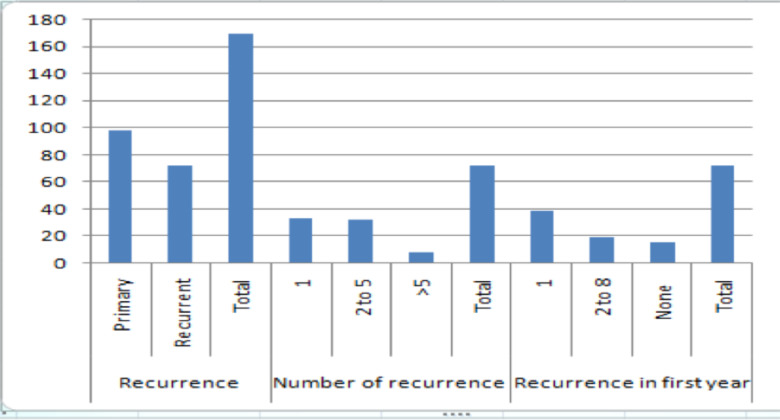
Pattern of recurrence of bladder tumor

Ninety-eight (57.6%) patients appeared with primary bladder tumors while 72 (42.4%) came with recurrent bladder tumor. Of the 72 patients 33 (45.8%) had a single recurrence, 32 (44.4%) had 2 to 5 recurrences and about 7 (9.8%) had more than 5 recurrences. When we see the first-year recurrences 15 (20.8%) had none, 38 (52.8%) had 1 recurrence and19 (26.4%) had 2 to8 recurrences (Figure 1).

Patients presented with hematuria 111 (65.3%), combined hematuria and lower urinary tract Symptoms (LUTS) 52 (30.6%), LUTS alone 4 (2.3%) and other symptoms like suprapubic pain and dysuria 3 (1.8%). Forty (23.5%) patients have abnormal (>1.2mg per dl) Creatinine. Ultrasonographic evaluation showed 95 (55.9%) bladder mass, 37 (21.8%) bladder mass with hydronephrosis and 38 (22.3%) other results like bladder wall thickening and normal results (Table 2).

**Table 2:** Characteristics of bladder tumor patients at presentation (n=170)

Variables and category		Frequency	percent
Symptoms	Hematuria	111	65.3
	Hematuria and LUTS	52	30.6
	LUTS	4	2.3
	Other	3	1.8
Creatinine	Normal (<1.2mg/dl)	130	76.5
	Abnormal (>1.2mg/dl)	40	23.5
Ultrasonography	Bladder mass	95	55.9
	Bladder mass and		
	hydronephrosis	37	21.8
	Other	38	22.3

Most of the recurrent tumors 55 (76.4%) had huge size and were multiple 62 (86.1%) in the primary presentation. Most recurrent tumors 47 (65.3%) are low grade bladder tumors. about 17 (23.6%) were high grade tumor in their primary presentation (Table 3).

**Table 3:** Tumor characteristics of recurrent bladder tumors (n=72)

Variable		Frequency	percent
Size	Huge	55	76.4
	Small	17	23.6
Multiplicity	Multiple	62	86.1
	Single	10	13.9
Grade	Low grade	47	65.3
	High grade	17	23.6
	UNLMP	7	9.7
	Not recorded	1	1.4

## Discussion

In our study bladder tumor is common in ages 40 to 60 years which is one to two decades younger age than the developed countries. Whereas bladder tumor is rare below the age of 40 years, in our study it accounts 11.2% (19, 20). Even though, our study is hospital based, like most other studies, bladder tumor predominates in males with a 3 to 1 ratio, approximately. The ratio is about 4 to 1 in one study in the United Kingdom (21) and similar ratio is described in another review (19). The majority (83.5%) of patents are from the three regions namely Oromia, Amhara and Addis Ababa. this could probably be due to the population size of the regions and their proximity to the study hospital (TASH).

Both primary and recurrent bladder tumor patients were admitted to the hospital with approximately 3: 2 ratios. This indicates recurrent bladder tumor is a significant proportion of bladder tumors that should be tackled with all means of surgical and medical management. Recurrence is an important issue in bladder tumor management in other centers as well. Of the 72 patients with recurrent bladder tumor 57 (79 %) have recurred within the first year and 19 (26.4%) had multiple recurrences within the first year. This shows bladder tumor is a highly recurrent tumor (12).

The main symptom of presentation in 163 (96%) of bladder tumor patients observed in our study is hematuria which similar to many other studies. This is followed by LUTS either combined with hematuria or LUTS alone and suprapubic pain (22, 23).

Upper urinary tract obstruction is one of the complications of bladder tumor. In our study a significant proportion of patients (n=40, 23 %) had elevated serum Creatinine (>1.2mg/dl). In addition, 40 patients (21.8%) had hydronephrosis on ultrasound during their presentation. Both elevated Creatinine and hydronephrosis show advanced bladder tumor. the presence of hydronephrosis has prognostic value in bladder cancer (24).

Most of the recurrent tumors are huge (76.4%) and multiple (86.1%) at their initial presentation. However, small and single tumors at initial presentations have also recurred in this study. Though many European studies showed size and multiplicity increase risk of recurrence, our patients have late presentations which probably made the proportion of recurrence higher in this group. It needs further prospective studies to make a conclusion (9,19).

In conclusions most bladder tumor patients present with hematuria and LUTS. Recurrent bladder tumors are significant portion of bladder tumor patients. Most of the recurrent bladder tumors have huge size and multiple in number at their initial presentation. All histological variants of urothelial carcinomas recur. We recommend a prospective study for better characterization of recurrent bladder tumors.
